# A nationwide observational cohort study of the relationship between beta-blockade and survival after hip fracture surgery

**DOI:** 10.1007/s00068-020-01588-7

**Published:** 2021-01-28

**Authors:** Rebecka Ahl, Ahmad Mohammad Ismail, Tomas Borg, Gabriel Sjölin, Maximilian Peter Forssten, Yang Cao, Per Wretenberg, Shahin Mohseni

**Affiliations:** 1 Division of Trauma and Emergency Surgery, Department of Surgery, Karolinska University Hospital, Stockholm, Sweden; 2 Division of Surgery, Department of Clinical Science Intervention and Technology (CLINTEC), Karolinska Institutet, Stockholm, Sweden; 3 School of Medical Sciences, Orebro University, Orebro, Sweden; 4 Department of Orthopedic Surgery, Orebro University Hospital, Orebro, Sweden; 5 Division of Trauma and Emergency Surgery, Department of Surgery, Orebro University Hospital, Orebro, Sweden; 6 Clinical Epidemiology and Biostatistics, School of Medical Sciences, Orebro University, Orebro, Sweden

**Keywords:** Hip fractures, Mortality, Beta-blockers

## Abstract

**Purpose:**

Despite advances in the care of hip fractures, this area of surgery is associated with high postoperative mortality. Downregulating circulating catecholamines, released as a response to traumatic injury and surgical trauma, is believed to reduce the risk of death in noncardiac surgical patients. This effect has not been studied in hip fractures. This study aims to assess whether survival benefits are gained by reducing the effects of the hyper-adrenergic state with beta-blocker therapy in patients undergoing emergency hip fracture surgery.

**Methods:**

This is a retrospective nationwide observational cohort study. All adults $\ge$ 18 years were identified from the prospectively collected national quality register for hip fractures in Sweden during a 10-year period. Pathological fractures were excluded. The cohort was subdivided into beta-blocker users and non-users. Poisson regression with robust standard errors and adjustments for confounders was used to evaluate 30-day mortality.

**Results:**

134,915 patients were included of whom 38.9% had ongoing beta-blocker therapy at the time of surgery. Beta-blocker users were significantly older and less fit for surgery. Crude 30-day all-cause mortality was significantly increased in non-users (10.0% versus 3.7%, *p* < 0.001). Beta-blocker therapy resulted in a 72% relative risk reduction in 30-day all-cause mortality (incidence rate ratio 0.28, 95% CI 0.26–0.29, *p* < 0.001) and was independently associated with a reduction in deaths of cardiovascular, respiratory, and cerebrovascular origin and deaths due to sepsis or multiorgan failure.

**Conclusions:**

Beta-blockers are associated with significant survival benefits when undergoing emergency hip fracture surgery. Outlined results strongly encourage an interventional design to validate the observed relationship.

**Supplementary Information:**

The online version contains supplementary material available at 10.1007/s00068-020-01588-7.

## Background

High early mortality following neck of femur and peritrochanteric fractures (collectively known as proximal femur fractures) places a heavyweight on the field of orthopedic surgery. These fractures are not only associated with high death rates, but postsurgical repercussions seriously affect public health. Early postoperative deaths range between 10 and 16% and overall mortality rates within one year of surgery reach approximately double [1, 2]. Outcomes are even worse in patients managed non-surgically, even accounting for fragility levels [3]. Proximal femur fractures are disproportionately high in geriatric patients. Thirty-four persons per 10,000 aged $\ge 50\text{ years}$ in Sweden suffered a proximal femur fracture in 2018. The mean age of this patient group was 82 years. [4] The corresponding incidence of geriatric hip fractures in the United States is 57 per 10,000 [5].

Mortality remains high in this patient group despite efforts to improve surgical care by deploying multidisciplinary care teams involving both geriatricians and orthopedic surgeons [6]. With a globally increasing geriatric population the frailty in hospitalized surgical patients is increasing. This may be one reason why mortality remains high in this patient group [7]. Other contributing factors may include the presence of concomitant cardiovascular and respiratory comorbidities which remain two of the commonest causes of in-hospital deaths following emergency surgery for proximal femur fractures [8, 9]. Consequently, proximal femur fractures represent a high economic burden for healthcare systems and society, necessitating the use of preventative or ameliorating interventions.

Beta-blockade has drawn increased attention in the past two decades as a possible means of reducing the risk of adverse events after noncardiac surgery [10, 11]. Major cardiac events are estimated to occur in ≈5% of patients undergoing noncardiac surgery [12] and patients with pre-existing cardiac risk factors are believed to benefit the most from beta-blocker protection [13]. In patients undergoing emergency orthopedic surgery, the number of major cardiac events is more than 10% [14]. Data suggest that beta-blockade is underused in noncardiac surgery [15] and that it has a prophylactic role in preventing postoperative adverse events, particularly cardiovascular deaths [10, 11]. There is a distinct gap in the literature concerning the use of beta-adrenergic blockade in patients undergoing orthopedic surgery for proximal femur fractures. This study investigates the relationship between beta-blockade and 30-day mortality in this patient group.

## Methods

### Data collection

This observational cohort study adheres to the principles of the Declaration of Helsinki. Ethical approval was obtained from the National Ethics Review Board (reference number 2019-02094). The national and prospectively collected patient register for proximal femur fractures “Rikshoft” was utilized to identify all adults aged $\ge 18\text{ years}$ undergoing primary surgery for a proximal femur fracture between January 2008 and December 2017 in Sweden. Patients with pathological fractures from primary or secondary bone tumors and those who had a re-operation were excluded. The Rikshoft register was created in the 1980s. It collects and maintains data on all proximal femur fractures suffered in Sweden [16]. Patient data obtained from the register included the date of hospital admission, age, sex, fracture type, American Society of Anesthesiologist (ASA) classification, Body Mass Index (BMI), date of surgery, surgical method, and hospital discharge date.

Data on postoperative mortality, comorbidity and beta-blocker use were obtained from national registers maintained by the National Board of Health and Welfare. Mortality data $\le 90\text{ days}$ postoperatively were collected from the Cause of Death register that records all deaths of Swedish residents and cause(s) of death stated on the death certificate. Comorbidity data were collected from the National Patient Register of both primary and secondary healthcare providers. This information was used to calculate a Charlson Comorbidity Index (CCI) for each patient. Finally, data on beta-blocker prescriptions (ACT codes C07AA, C07AB, C07AG) were attained from the national drug register. The national drug register is a population-based database that records all drug prescriptions issued by physicians in Sweden. Beta-blocker data were obtained within 1 year before and after surgery. This was chosen since beta-blockers rarely are discontinued once initiated and, therefore, commonly issued on a long-term basis covering up to a one-year period with a single prescription. Patients were included as beta-blocker users (BB^+^) if they were issued and collected a beta-blocker prescription. All other patients were considered beta-blocker non-users (BB^−^). Patients on beta-blocker therapy were assumed to continue beta-blocker intake during their hospital admission. Due to the lack of access to nationwide hospital records, this assumption was tested by assessing the electronic medical records of all patients included from one selected county during a specified time-period.

### Analysis

The primary outcome of interest was 30-day all-cause mortality following surgery for proximal femur fractures. The secondary outcome of interest was cause-specific mortality. Patient demographics and clinical outcomes were compared between BB^+^ and BB^−^ groups. Statistical tests included Chi-square test for categorical variables and Student’s *t* test (or Mann–Whitney $U$ test where appropriate) for continuous variables. The relationship between beta-blocker exposure and 30-day all-cause and cause-specific mortality was evaluated using Poisson regression with robust standard errors of variance [17]. Potential confounding was accommodated in the models by including age, sex, ASA, CCI, type of fracture and surgical method. The time from surgery to death or to the end of 30-day follow-up was used as an offset in the Poisson regression models. The outcome measure was reported as Incidence Rate Ratio (IRR) with 95% confidence interval (CI). Multiple imputations were used to handle missing values [18]. Results were considered statistically significant at a two-sided *p* value of < 0.05.

## Results

**Fig. 1:**
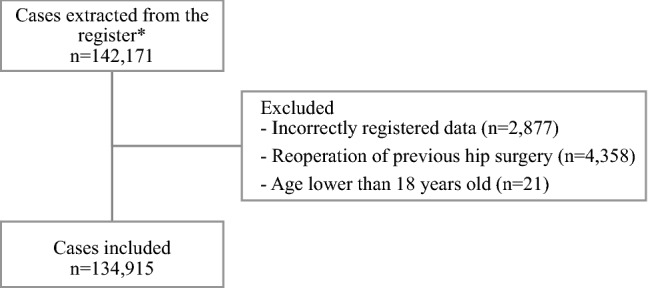
Flow chart describing patient selection. *Pathological fractures were not extracted

During the 10-year inclusion period, a total of 134,915 cases were identified. The total number of cases before exclusions was 142,171 (see Fig. 1 for patient selection). Inclusion coverage for the Rikshoft register in the studied period was $>90\%$ [16]. A total of 38.9% of the study cohort had a regular beta-blocker agent prescribed, of which 89.6% were selective beta-blocking agents. This degree of beta-blocker use is in line with the Swedish population aged $\ge 65\text{ years}$ for the same time-period. The corresponding rate for the Swedish population aged $\ge 18 \;years$ is under 15% [19]. The most common choice of beta-blocker agent was metoprolol ($>50\%$ of prescriptions). To assess adherence to continued beta-blocker treatment during hospital admission, in-hospital medical records of 2443 patients (1.8% of the total cohort) were reviewed. This subgroup represents all adults undergoing surgery for a proximal femur fracture at Örebro University Hospital and two county hospitals in Örebro County between January 2013 and December 2017. Nine hundred (36.8%) of these patients were prescribed beta-blocker therapy, none of whom were discontinued on their regular beta-blocker during their hospital stay.

There were differences between BB^+^ and BB^−^ groups. BB^+^ patients had a slight increase in mean [standard deviation] age: 83 [9] years compared to 81 [11] years ($p$ < 0.001) and a lower percentage of men: 30.0% ($n = \mathrm{15,738}$) versus 33.1% ($n=\mathrm{27,250}$) ($p$ < 0.001). Beta-blocker users were considered to be of higher surgical risk with higher ASA classifications and CCI scores (Table 1). The distribution of fracture types and surgical fixation methods differed significantly between groups. However, displaced cervical hip fractures were the commonest fractures in both groups. The most common choices of surgical fixation were sideplates and hemiarthroplasty (Table 1). Beta-blocker users were typically more burdened by medical conditions. There were statistically significant differences between the groups for all conditions except liver disease. Cardiovascular disease, cerebrovascular disease, chronic obstructive pulmonary disease, diabetes, connective tissue disease, peptic ulcer disease, chronic kidney disease, and cancer were all more common in BB^+^ patients (Table 2). As expected, the most significant difference was found in cardiovascular diseases. Hypertension, previous myocardial infarction, arrythmia and congestive heart failure were all observed much more frequently in BB^+^ patients (Table 2). Conversely, dementia and metastatic cancer were more common in BB^−^ patients: 22.8% in BB^−^ versus 16.1% in BB^+^ (*p* < 0.001) and 2.5% in BB^−^ versus 1.8% in BB^+^ (*p* < 0.001), respectively.

**Table 1:** Demographics, clinical characteristics and outcomes in beta-blocker non-users (BB^−^) and beta-blocker users (BB^+^) undergoing hip fracture surgery

Variable	Total *N* = 134,915	BB^−^ *N* = 82,415	BB^+^ *N* = 52,500	*p*
Age in years, mean (SD)	82.0 (10.0)	81.4 (10.8)	82.8 (8.5)	< 0.001
Sex, *n* (%)				< 0.001
Female	91,913 (68.1)	55,158 (66.9)	36,755 (70.0)	
Male	42,988 (31.9)	27,250 (33.1)	15,738 (30.0)	
Missing	14 (0.0)	7 (0.0)	7 (0.0)	
Type of Beta-blocker, *n* (%)				N/A
Metoprolol	30,143 (22.3)	–	30,143 (57.4)	
Bisoprolol	9805 (7.3)	–	9805 (18.7)	
Atenolol	7094 (5.3)	–	7094 (13.5)	
Other	5458 (4.0)	–	5458 (10.4)	
ASA* classification, *n* (%)				< 0.001
1	6656 (4.9)	5775 (7.0)	881 (1.7)	
2	48,264 (35.8)	32,136 (39.0)	16,128 (30.7)	
3	66,857 (49.6)	37,170 (45.1)	29,687 (56.5)	
4	10,534 (7.8)	5701 (6.9)	4833 (9.2)	
5	135 (0.1)	89 (0.1)	46 (0.1)	
Missing	2469 (1.8)	1544 (1.9)	925 (1.8)	
CCI^#^, *n* (%)				< 0.001
≤ 4	59,611 (44.2)	40,162 (48.7)	19,449 (37.0)	
5–6	50,247 (37.2)	29,705 (36.0)	20,542 (39.1)	
≥ 7	25,057 (18.6)	12,548 (15.2)	12,509 (23.8)	
Fracture type, *n* (%)				< 0.001
Non-displaced cervical (Garden 1–2)	17,868 (13.2)	11,560 (14.0)	6,308 (12.0)	
Displaced cervical (Garden 3–4)	50,172 (37.2)	30,518 (37)	19,654 (37.4)	
Basicervical	4480 (3.3)	2799 (3.4)	1681 (3.2)	
Peritrochanteric (two fragments)	26,859 (19.9)	16,191 (19.6)	10,668 (20.3)	
Peritrochanteric (multiple fragments)	24,493 (18.2)	14,785 (17.9)	9708 (18.5)	
Subtrochanteric	10,988 (8.1)	6533 (7.9)	4455 (8.5)	
Missing	55 (0.0)	29 (0.0)	26 (0.0)	
Type of surgery, *n* (%)				< 0.001
Pins or screws	23,458 (17.4)	15,291 (18.6)	8167 (15.6)	
Screws or pins with sideplate	34,902 (25.9)	21,450 (26.0)	13,452 (25.6)	
Intramedullary nail	31,992 (23.7)	18,968 (23.0)	13,024 (24.8)	
Hemiarthroplasty	34,596 (25.6)	20,574 (25.0)	14,022 (26.7)	
Total hip replacement	9889 (7.3)	6080 (7.4)	3809 (7.3)	
Missing	78 (0.1)	52 (0.1)	26 (0.0)	

^*^American Society of Anesthesiologists

^#^Charlson comorbidity index

**Table 2:** Preoperative co-morbidities in beta-blocker non-users (BB^−^) and beta-blocker users (BB^+^) undergoing hip fracture surgery

		BB^−^ *N* = 82,415	BB^+^ *N* = 52,500	*P*
Myocardial infarction, *n* (%)		2567 (3.1)	5496 (10.5)	< 0.001
Hypertension, *n* (%)		23,454 (28.5)	28,302 (53.9)	< 0.001
Arrythmia, *n* (%)		8479 (10.3)	16,519 (31.5)	< 0.001
Congestive heart failure, *n* (%)		8252 (10.0)	12,845 (24.5)	< 0.001
Peripheral vascular disease, *n* (%)		2849 (3.5)	3,041 (5.8)	< 0.001
Cerebrovascular event, *n* (%)		12,533 (15.2)	10,849 (20.7)	< 0.001
Dementia, *n* (%)		18,827 (22.8)	8477 (16.1)	< 0.001
Chronic obstructive pulmonary disease, *n* (%)		9123 (11.1)	6454 (12.3)	< 0.001
Connective tissue disease, *n* (%)		3430 (4.1)	3057 (5.8)	< 0.001
Peptic ulcer disease, *n* (%)		2372 (2.9)	1956 (3.7)	< 0.001
Liver disease, *n* (%)				
Mild		583 (0.7)	438 (0.8)	0.01
Severe		200 (0.2)	149 (0.3)	0.16
Diabetes mellitus, *n* (%)				
Uncomplicated		6508 (7.9)	6730 (12.8)	< 0.001
End-organ damage		3097 (3.8)	3521 (6.7)	< 0.001
Hemiplegia, *n* (%)		1560 (1.9)	1351 (2.6)	< 0.001
Chronic kidney disease, *n* (%)		2927 (3.6)	4018 (7.7)	< 0.001
Cancer, *n* (%)				
Local tumor		8620 (10.5)	5940 (11.3)	< 0.001
Metastatic		2025 (2.5)	937 (1.8)	< 0.001

Crude mortality was significantly higher for both 30-day (10.0% in BB^−^ versus 3.7% in BB^+^, *p* < 0.001) and 90-day (16.4% in BB^−^ versus 8.3% in BB^+^, *p* < 0.001) all-cause mortality in non-users despite our data showing a higher burden of preoperative disease, increased mean age and higher preoperative risk profiles for beta-blocker users (Table 1, 2 and 3). BB^+^ patients had longer hospital stays with a median [interquartile range] of 8 [5, 12] days compared to 7 [5, 11] days for non-users ($p$ < 0.001). On analysis of cause-specific deaths, cardiovascular events, respiratory failure and multiorgan failure were the most common causes of death for both BB^+^ and BB^−^ patients. A larger percentage of patients suffered cardiovascular deaths in the BB^−^ group (4.0% in BB^−^ versus 1.7% of the total BB^+^ group, $p$ <0.001). However, cardiovascular deaths represented a larger percentage of 30-day mortality in BB^+^ compared to BB^−^ patients (45.3% versus 40.0%, $p$ < 0.001) (Table 3). Regression analysis associated beta-blocker therapy with a 72% risk reduction in the incidence of 30-day all-cause mortality (IRR 0.28, 95% CI 0.26–0.29, $p$ < 0.001). Increasing age, ASA classification, and CCI scores were all predictive of a greater incidence of a fatal outcome (Table 4). Beta-blocker use was independently associated with a reduction in deaths of cardiovascular, respiratory and cerebrovascular origins as well as deaths due to sepsis and multiorgan failure, ranging between 65 and 77% (Table 5).

**Table 3:** Outcomes in beta-blocker non-users (BB^−^) and beta-blocker users (BB^+^) after hip fracture surgery

	BB^−^ *N* = 82,415	BB^+^ *N* = 52,500	*P*
Hospital length of stay, days			
Median (Q2, Q3)	7 (5, 11)	8 (5, 12)	< 0.001
30-day all-cause mortality, *n* (%)	8275 (10.0)	1933 (3.7)	< 0.001
90-day all-cause mortality, *n* (%)	13,512 (16.4)	4353 (8.3)	< 0.001
30-day cause-specific mortality*			
Cardiovascular event, *n* (%)	3307 (40.0)	875 (45.3)	< 0.001^*#*^
Respiratory failure, *n* (%)	1431 (17.3)	271 (14.0)	< 0.001^*#*^
Cerebrovascular event, *n* (%)	135 (1.6)	31 (1.6)	1.000^*#*^
Sepsis, *n* (%)	158 (1.9)	34 (1.8)	0.730^*#*^
Multiorgan failure, *n* (%)	2945 (35.6)	644 (33.3)	0.063^*#*^
Unknown, *n* (%)	299 (3.6)	78 (4.0)	0.410^*#*^

*Percentages calculated as fractions of the total number of 30-day deaths for the relevant subgroup

^#^Adjusted for multiple comparisons

**Table 4:** Incidence rate ratio (IRR) for 30-day mortality after hip fracture surgery

Variable		IRR (95% CI)	P
Beta-blocker therapy			
No		Ref	
Yes		0.28 (0.26–0.29)	< 0.001
Age		1.07 (1.06–1.07)	< 0.001
Sex			
Female		Ref	
Male		1.75 (1.68–1.83)	< 0.001
Charlson comorbidity index			
≤ 4		Ref	
5–6		1.81 (1.71–1.91)	< 0.001
≥ 7		2.87 (2.71–3.05)	< 0.001
ASA classification			
1		Ref	
2		1.35 (1.11–1.64)	0.002
3		2.53 (2.09–3.07)	< 0.001
4		5.24 (4.34–6.38)	< 0.001
5		11.95 (8.32–17.18)	< 0.001
Fracture type			
Non-displaced cervical (Garden 1–2)		Ref	
Displaced cervical (Garden 3–4)		1.44 (1.31–1.58)	< 0.001
Basicervical		1.35 (1.14–1.60)	< 0.001
Peritrochanteric (two fragments)		1.38 (1.17–1.61)	< 0.001
Peritrochanteric (multiple fragments)		1.46 (1.24–1.72)	< 0.001
Subtrochanteric		1.49 (1.26–1.77)	< 0.001
Type of surgery			
Screws or pins		Ref	
Screws or pins with sideplate		0.93 (0.80–1.07)	0.305
Intramedullary rod		0.89 (0.76–1.04)	0.127
Hemiarthroplasty		0.96 (0.88–1.04)	0.319
Total hip replacement		0.54 (0.46–0.63)	< 0.001

Poisson regression model with robust standard errors. Multiple imputation method for missing values. Model adjusted for age, sex, Charlson comorbidity index, ASA classification, fracture type and type of surgery

**Table 5:** The effect of beta-blocker therapy on the incidence rate ratio (IRR) for 30-day cause-specific mortality after hip fracture surgery

Mortality cause	IRR (95% CI)	*p* value
Cardiovascular*	0.24 (0.22–0.26)	< 0.001
Respiratory	0.23 (0.21–0.27)	< 0.001
Cerebrovascular	0.30 (0.20–0.44)	< 0.001
Sepsis	0.25 (0.17–0.37)	< 0.001
Multi-organ failure	0.25 (0.23–0.28)	< 0.001
Unknown	0.35 (0.26–0.46)	< 0.001

Model adjusted for age, sex, Charlson comorbidity index, ASA classification, fracture type and type of surgery

*Also adjusted for prior myocardial infarction, heart failure, and peripheral vascular disease

Only 5.9% (*n* = 7981) of the total cohort were under 65 years. Analyses were repeated to include patients aged 65 or above only. This corresponded to 94.1% (*n* = 126,934) of the initial total cohort. Beta-blocker use was registered in 39.9% (*n* = 50,683) of this geriatric subgroup. Crude mortality remained higher in non-users compared to beta-blocker users (10.7% versus 3.8%, *p* < 0.001) and regression analysis revealed a similar relative risk reduction in 30-day mortality by 69% for patients on beta-blocker therapy (IRR 0.31, 95% CI 0.29–0.32, *p* < 0.001) (see supplementary document for these tables).

## Discussion

Orthopedic trauma and subsequent emergency surgery represent an extreme physiological stress, especially for the geriatric body. Geriatric patients are overrepresented among patients with hip fractures which is reflected in the high mortality associated with such surgery. Results of this study support these statements with most patients in our nationwide cohort being ≈80 years (only 5.9% ($n = 7981/\mathrm{134,915}$) were under 65 years) and with an overall 30-day mortality of 7.6% ($n = \mathrm{10,208}$). Discussions have been ongoing for many years as to whether hip fractures are best managed via hemiarthroplasty or total hip arthroplasty. Recently, in a publication by Rogemark, trauma and orthopedic surgeons were encouraged to raise their heads “from a detailed comparison of two well-functioning surgical procedures and take more responsibility for the entire clinical pathway” of hip fracture patients [20]. Recognition of other patient factors such as cardiac comorbidities, frailty and age, is thus necessary [21].

Surgical trauma and anesthesia initiate a snowballing cascade of events including cardiovascular hyperactivity, hypercoagulability and inflammatory cell mobilization [12]. Circulating catecholamines and cortisol represent underlying forces. The downregulation of the catecholamine mediated effects by beta-receptor-blockade is believed to achieve beneficial outcomes in noncardiac surgery [22]. In this nationwide cohort study, we outline a significant survival benefit in the first 30 days after emergency orthopedic surgery for patients who are prescribed regular preoperative beta-blocker therapy. Our results outline an absolute risk difference of 6.3% and the number needed to treat for one patient to benefit is 16. The apparent protective relationship between beta-blockade and survival after emergency surgery for hip fractures was persistently observed in both crude data and after regression analysis. Interestingly, crude mortality was lower for beta-blocker users at both 30 and 90 days after surgery, despite the significantly higher age, ASA classification and CCI scores of this group.

### Cardiovascular mortality

Cardiovascular deaths are of specific relevance in the context of adrenergic overload as a result of tissue injury and subsequent major surgery [12]. Hip surgery has been especially associated with increased risk of major cardiac events in the postoperative period occurring in six percent of patients [14]. Adverse cardiac events during the immediate postoperative period does not only affect in-hospital survival but has a major influence on the continued mortality risk for several months after noncardiac surgery [23]. Therefore, to achieve reductions in postoperative mortality, focus must be directed towards the optimization of this high-risk group. Raw data in this study portray cardiovascular events as a crucial cause of death with a total of 4182 cardiovascular fatalities, corresponding to 41.0% of deaths in all patients. Beta-blocker use caused a 76% risk reduction in the incidence of cardiovascular deaths (IRR 0.24, 95% CI 0.22–0.26, *p* < 0.001). Similarly, the PeriOperative ISchemic Evaluation (POISE) trial published in 2008 showed a significant reduction in adverse cardiac events in noncardiac surgical patients treated with metoprolol [24]. The degree of benefit appears to increase with increasing cardiac risk index [25].

Furthermore, two meta-analyses and a Cochrane review published in the last 15 years on beta-blocker therapy and noncardiac surgery have outlined protective effects on adverse cardiac events [11, 26, 27]. Consequently, international guidelines from the American Heart Association, American College of Cardiology (AHA/ACC) and the European Society of Cardiology (ESC) both advise against discontinuing regular beta-blockers prior to or following any surgery [28, 29]. The results of this nationwide study are in line with these recommendations and support the use of beta-blockers in orthopedic patients undergoing emergency surgery for hip fractures to prevent postoperative cardiac deaths. Interestingly, 55.3% (*n* = 45,601) of BB^−^ patients had a diagnosis of cardiovascular disease prior to admission. In particular, congestive heart failure was found in 10.0% (*n* = 8,252) and arrythmia in 10.3% (*n* = 8,479) of patients in the BB^−^ group, suggesting that there may be a substantial underuse of beta-blockade in this patient group.

### Non-cardiac mortality

The Agency for Healthcare Research and Quality’s evidence report from 2001 promotes the use of perioperative beta-blocker therapy as the second-highest-rated safety practice protecting against surgical mortality [30]. In 2013, an updated version of the report amended these recommendations, stating that perioperative beta-blockers should not be considered a safe practice for all surgical patients [31]. The revision was to a large extent based on the observation of an increase in all-cause mortality and non-fatal strokes from recent randomized trials [24, 26]. In contrast, the results of the current study detect a significant reduction in 30- and 90-day all-cause mortality and a survival benefit that was doubled when comparing beta-blocker users to non-users irrespective of which cause of death analyzed. Although limited by the retrospective nature, the authors hypothesize that this observed pattern could be the result of a widespread benefit of a general antagonistic action to catecholamine hyperactivation in a patient group with high general frailty whose physiology is inadequate to safely deal with uncontrolled adrenergic storms.

There are other methodological differences which can explain this difference. Most scientific work on beta-blockers in noncardiac surgery is based on a mixture of noncardiac surgery. For instance, the POISE trial included vascular, orthopedic and abdominal surgery. While this may lead to increased generalizability, it increases the risk of including undetected biases. To the best knowledge of the authors, our group is the first to evaluate beta-blockers in fracture surgery, specifically. Furthermore, the POISE trial administered a high dose of extended-release metoprolol (200 mg) to beta-blocker naive patients with drug administration occurring only hours prior to surgery. These decisions reflect a major difference to the current study which evaluates a cohort with a narrow age span, undergoing surgery for the same indication, and with most patients being prescribed 50 mg of extended-release metoprolol. The authors hypothesize that these methodological modifications may have led to fewer bradycardic/hypotensive episodes and thereby avoided the mechanisms believed to underlie previously observed increased stroke risks. Our results demonstrate a 70% risk reduction in fatal strokes for beta-blocker users (IRR 0.30, 95% CI 0.20–0.44, *p* < 0.001). Similar results have been reported by Obeid et al. who demonstrated a 34% reduction in stroke/death risk in beta-blocker treated patients undergoing vascular surgery [32].

The literature on beta-blockers in noncardiac surgery is growing and there is increasing belief that catecholamines play an imperative role in the initiation of inflammatory responses and subsequent complications and resulting multiorgan failure [33]. Regression analysis in the current study demonstrate a significant reduction in deaths attributable to respiratory causes, sepsis and multiorgan failure (Table 5). The stress response from tissue injury results in the release of cytokines interleukin (IL)-1, IL-6 and tumor necrosis factor-α [22]. Modulating immune responses by decreasing cytokine release through beta-blockers has been reported in animal models of traumatic injury [34, 35]. Cytokine downregulation is one proposed mechanism explaining reports of survival advantages for beta-blocker exposed patients treated for severe sepsis and burns [36, 37]. In animal models of pulmonary dysfunction, beta-blockade has demonstrated attenuation of lung injury and acute respiratory distress syndromes [38]. These mechanisms could explain the reduction in the deaths due to sepsis, pulmonary events and multiorgan failure seen in beta-blocker users in the current study. Regrettably, due to a high proportion of missing data in postoperative complications in the Rikshoft register, we are unable to account for the incidence of non-fatal complications. This is a recognized limitation and we would encourage this to be addressed in a randomized trial.

Furthermore, due to the use of national quality registers we cannot with certainty conclude that all BB^+^ patients received continued beta-blocker therapy after hospital admission. However, a review of 2443 patients from three hospitals in Örebro County demonstrates that discontinuation of beta-blocker treatment does not normally occur. The continuation of a regular beta-blocker whilst in hospital is also clearly enforced by both American and European cardiology guidelines [28, 29]. In contrast to major abdominal surgery, orthopedic patients do not generally have any restrictions regarding enteral nutrition postoperatively, which further reduces the risk of in-hospital discontinued drug administrations.

## Conclusions

The current study outlines a significant survival benefit for patients on regular beta-blockers who suffer a hip fracture and undergo emergency orthopedic surgery. While we are unable to conclude anything about causal pathways, the results suggest a causal relationship and should strongly encourage strict adherence to prescribed beta-blocker treatment for the duration of the hospital stay, and urge preoperative cardiac risk assessments to identify beta-blocker naive patients meeting criteria for beta-blockers but lacking treatment. The results suggest that beta-blocker therapy is widely underused for patients with hip fractures. In addition, the authors believe that these results may help pave the way for future interventional trials and improve quality of care for patients undergoing hip fracture surgery.

## Supplementary Information

Below is the link to the electronic supplementary material.

Supplementary file1 (DOCX 25 KB)

## Data Availability

May be made available on reasonable request provided the appropriate ethical approval is sought and approved.
