# Supplementary material for: A nationwide observational cohort study of the relationship between beta-blockade and survival after hip fracture surgery
Source: Eur J Trauma Emerg Surg. 2021 Jan 28;48(2):743–51. doi: 10.1007/s00068-020-01588-7 (PMC9001555; doi:10.1007/s00068-020-01588-7)
Supplement: Supplementary file 1 — Supplementary file1 (DOCX 25 KB) [file 68_2020_1588_MOESM1_ESM.docx]

| Table 6. Demographics, clinical characteristics and outcomes in beta-blocker non-users (BB^-^) and beta-blocker users (BB^+^) *over 65 years* undergoing hip fracture surgery | | | | |
| --- | --- | --- | --- | --- |
| **Variable** | **Total**  **N = 126,934** | **BB^-^**  **N = 76,251** | **BB^+^**  **N = 50,683** | **p** |
| **Age in years, mean [SD]** | 83.6 (±7.7) | 83.5 (±7.9) | 83.7 (±7.3) | < 0·001 |
| **Sex, n (%)** | | | | < 0·001 |
| Female | 87,933 (69.3) | 52,050 (68.3) | 35,883 (70.8) |  |
| Male | 38,987 (30.7) | 24,194 (31.7) | 14,793 (29.2) |  |
| Missing | 14 (0.0) | 7 (0.0) | 7 (0.0) |  |
| **Type of Beta-blocker, n (%)** | | | | N/A |
| Metoprolol | 29,106 (22.9) | - | 29,106 (22.9) |  |
| Bisoprolol | 9,551 (7.5) | - | 9,551 (7.5) |  |
| Atenolol | 6,866 (5.4) | - | 6,866 (5.4) |  |
| Other | 5,160 (4.1) | - | 5,160 (4.1) |  |
| **ASA* classification, n (%)** | | | | < 0·001 |
| 1 | 4,926 (3.9) | 4,148 (5.4) | 778 (1.5) |  |
| 2 | 44,884 (35.4) | 29,512 (38.7) | 15,372 (30.3) |  |
| 3 | 64,527 (50.8) | 35,616 (46.7) | 28,911 (57.0) |  |
| 4 | 10,159 (8.0) | 5,473 (7.2) | 4,686 (9.2) |  |
| 5 | 132 (0.1) | 87 (0.1) | 45 (0.1) |  |
| Missing | 2,306 (1.8) | 1,415 (1.9) | 891 (1.8) |  |
| **CCI^#^, n (%)** | | | | < 0·001 |
| ≤4 | 52,581 (41.4) | 34,549 (45.3) | 18,032 (35.6) |  |
| 5-6 | 49,681 (39.1) | 29,382 (38.5) | 20,299 (40.1) |  |
| ≥7 | 24,672 (19.4) | 12,320 (16.2) | 12,352 (24.4) |  |
| **Fracture type, n (%)** | | | | < 0·001 |
| Non-displaced cervical (Garden 1-2) | 16,224 (12.8) | 10,259 (13.5) | 5,965 (11.8) |  |
| Displaced cervical (Garden 3-4) | 47,344 (37.3) | 28,330 (37.2) | 19,014 (37.5) |  |
| Basicervical | 4,153 (3.3) | 2,537 (3.3) | 1,616 (3.2) |  |
| Peritrochanteric (two fragments) | 25,512 (20.1) | 15,167 (19.9) | 10,345 (20.4) |  |
| Peritrochanteric (multiple fragments) | 23,495 (18.5) | 14,039 (18.4) | 9,456 (18.7) |  |
| Subtrochanteric | 10,153 (8.0) | 5,892 (7.7) | 4,261 (8.4) |  |
| Missing | 53 (0.0) | 27 (0.0) | 26 (0.1) |  |
| **Type of surgery, n (%)** | | | | < 0·001 |
| Pins or screws | 20,231 (15.9) | 12,729 (16.7) | 7,502 (14.8) |  |
| Screws or pins with sideplate | 32,960 (26.0) | 19,947 (26.2) | 13,013 (25.7) |  |
| Intramedullary nail | 30,333 (23.9) | 17,714 (23.2) | 12,619 (24.9) |  |
| Hemiarthroplasty | 34,321 (27.0) | 20,380 (26.7) | 13,941 (27.5) |  |
| Total hip replacement | 9,016 (7.1) | 5,433 (7.1) | 3,583 (7.1) |  |
| Missing | 73 (0.1) | 48 (0.1) | 25 (0.0) |  |

* American Society of Anesthesiologists, # Charlson Comorbidity Score

| Table 7. Outcomes in beta-blocker non-users (BB^-^) and beta-blocker users (BB^+^) *over 65 years* after hip fracture surgery | | | | |
| --- | --- | --- | --- | --- |
|  |  | **BB^-^** | **BB^+^** |  |
|  |  | **N = 76,251** | **N = 50,683** | **p** |
| Hospital length of stay, days | | |  |  |
|  | Median [Q2,Q3] | 7 [5,11] | 9 [5,12] | <0·001 |
| 30-day all-cause mortality, n (%) | | 8,179 (10.7) | 1,922 (3.8) | <0·001 |
| 90-day all-cause mortality, n (%) | | 13,321 (17.5) | 4,325 (8.5) | <0·001 |
| 30-day cause-specific mortality* | | |  |  |
|  | Cardiovascular event, n (%) | 3,281 (40.1) | 871 (45.3) | <0.001*^#^* |
|  | Respiratory failure, n (%) | 1,418 (17.3) | 267 (13.9) | <0.001*^#^* |
|  | Cerebrovascular event, n (%) | 134 (1.6) | 31 (1.6) | 1.000*^#^* |
|  | Sepsis, n (%) | 156 (1.9) | 34 (1.8) | 0.760*^#^* |
|  | Multiorgan failure, n (%) | 2,897 (35.4) | 642 (33.4) | 0.010*^#^* |
|  | Unknown, n (%) | 233 (2.8) | 65 (3.4) | 0.240*^#^* |
| ** Percentages calculated as fractions of the total number of 30-day deaths for the relevant subgroup*  *^#^Adjusted for multiple comparisons.* | | | | |

| Table 8. Incidence rate ratio (IRR) for 30-day mortality after hip fracture surgery in patients *over 65 years* | | | | |
| --- | --- | --- | --- | --- |
| Variable |  | **IRR (95% CI)** | **p** |  |
| Beta-blocker therapy | |  |  |  |
|  | No | ref. |  |  |
|  | Yes | 0·31 (0·29-0·32) | <0·001 |  |
| Age |  | 1·06 (1·05-1·06) | <0·001 |  |
| Sex |  |  |  |  |
|  | Female | ref. |  |  |
|  | Male | 1·68 (1·61-1·75) | <0·001 |  |
| Charlson Comorbidity Index | | |  |  |
|  | ≤4 | ref. |  |  |
|  | 5-6 | 1·72 (1·63-1·83) | <0·001 |  |
|  | ≥7 | 2·55 (2·40-2·72) | <0·001 |  |
| ASA Classification | |  |  |  |
|  | 1 | ref. |  |  |
|  | 2 | 1·27 (1·04-1.55) | 0·019 |  |
|  | 3 | 2·33 (1·92-2·84) | <0·001 |  |
|  | 4 | 4·31 (3·52-5·27) | <0·001 |  |
|  | 5 | 7·58 (5·43-10·58) | <0·001 |  |
| Fracture type | |  |  |  |
|  | Non-displaced cervical (Garden 1-2) | ref. |  |  |
|  | Displaced cervical (Garden 3-4) | 1·34 (1·22-1·48) | <0·001 |  |
|  | Basicervical | 1·27 (1·07-1·50) | <0·001 |  |
|  | Peritrochanteric (two fragments) | 1·27 (1·09-1·49) | <0·001 |  |
|  | Peritrochanteric (multiple fragments) | 1·38 (1·17-1·61) | <0·001 |  |
|  | Subtrochanteric | 1·40 (1·18-1·66) | <0·001 |  |
| Type of surgery | |  |  |  |
|  | Screw or pin | ref. |  |  |
|  | Screws or pins with sideplate | 0·95 (0·82-1·10) | 0·501 |  |
|  | Intramedullary rod | 0·90 (0·77-1·05) | 0·184 |  |
|  | Hemiarthroplasty | 0·96 (0·89-1·04) | 0·366 |  |
|  | Total hip replacement | 0·59 (0·50-0·69) | <0·001 |  |
| *Poisson regression model with robust standard errors. Multiple imputation method for missing values. Model adjusted for age, sex, Charlson comorbidity index, ASA classification, fracture type and type of surgery.* | | | | |
